# Correction: Transfer learned potential energy surfaces: accurate anharmonic vibrational dynamics and dissociation energies for the formic acid monomer and dimer

**DOI:** 10.1039/d2cp90126a

**Published:** 2022-07-19

**Authors:** Silvan Käser, Markus Meuwly

**Affiliations:** Department of Chemistry, University of Basel Klingelbergstrasse 80 CH-4056 Basel Switzerland m.meuwly@unibas.ch

## Abstract

Correction for ‘Transfer learned potential energy surfaces: accurate anharmonic vibrational dynamics and dissociation energies for the formic acid monomer and dimer’ by Silvan Käser *et al.*, *Phys. Chem. Chem. Phys.*, 2022, **24**, 5269–5281, https://doi.org/10.1039/D1CP04393E.

The authors would like to make a correction to Fig. 1 and the corresponding caption where the values for the axes are assigned incorrectly to the data in the published article. The corrected figure and figure caption are shown below.

**Fig. 1:**
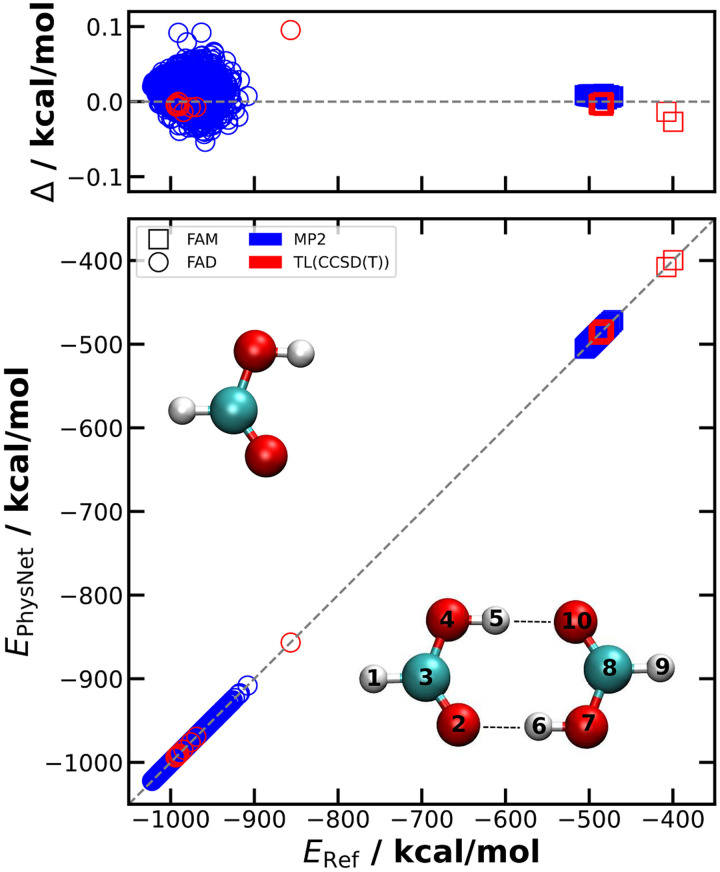
The accuracy of the PES_MP2_ and PES_TL_ energies is shown with respect to the appropriate reference *ab initio* values taken from the separate test sets. For PES_MP2_, only the performance of the superior model is shown and only FAM and FAD geometries of the test set are considered. The deviations *Δ* = *E*_Ref_ − *E*_PhysNet_ for geometries in the test sets remain below 0.1 kcal mol^−1^. The structures of FAM and FAD are shown together with labels and the hydrogen bonds indicated by dotted lines.

The Royal Society of Chemistry apologises for these errors and any consequent inconvenience to authors and readers.

## Supplementary Material

